# Skin Diseases of Children

**Published:** 1898-02

**Authors:** 


Skin Diseases of Children. By George Henry Fox, A. M., M. D., Clinical Professor of Diseases of the Skin, College of Physicians and Surgeons, New York; Physician to the New York Skin and Cancer Hospital, etc. With twelve photogravure and chromographic plates and sixty illustrations in the text. New York: William Wood & Company. 1897.

The volume before us deals with those diseases of the skin most frequently met with in infancy and early life. The first portion of the work is occupied by a concise and careful description of the maladies which so many times cause sudden alarm in a household and which the busy practitioner is so often called upon to diagnosticate and treat off-hand, to decide whether that signal of distress be well or ill-founded, whether the difficulty is or is not contagious.

The symptomatology of the individual articles all strike the reader at once as being clear, quite complete and thoroughly modern, while the treatment is eminently practical and fully considered under the heading of each disease, but for greater convenience of reference and comparison, remedies and methods of treatment are formulated and systematised in the concluding portion of the work.

As to the photogravure and chromographic plates, we have nothing to say that is not laudatory ; the plates illustrating alopecia areata, ichthyosis and syphilis hereditaria are, in our opinion, the gems of the book. E. W.

